# Affordance embeddings for situated language understanding

**DOI:** 10.3389/frai.2022.774752

**Published:** 2022-09-23

**Authors:** Nikhil Krishnaswamy, James Pustejovsky

**Affiliations:** ^1^Situated Grounding and Natural Language Lab, Department of Computer Science, Colorado State University, Fort Collins, CO, United States; ^2^Lab for Linguistics and Computation, Department of Computer Science, Brandeis University, Waltham, MA, United States

**Keywords:** situated grounding, multimodal dialogue, neurosymbolic intelligence, affordance learning, embodiment, interactive agents, VoxML

## Abstract

Much progress in AI over the last decade has been driven by advances in natural language processing technology, in turn facilitated by large datasets and increased computation power used to train large neural language models. These systems demonstrate apparently sophisticated linguistic understanding or generation capabilities, but often fail to transfer their skills to situations they have not encountered before. We argue that computational situated grounding of linguistic information to real or simulated scenarios provide a solution to some of these learning challenges by creating situational representations that both serve as a formal model of the salient phenomena, and contain rich amounts of exploitable, task-appropriate data for training new, flexible computational models. We approach this problem from a *neurosymbolic* perspective, using multimodal contextual modeling of interactive situations, events, and object properties, particularly *afforded* behaviors, and *habitats*, the situations that condition them. These properties are tightly coupled to processes of situated grounding, and herein we discuss we combine neural and symbolic methods with multimodal simulations to create a platform, VoxWorld, for modeling communication in context, and we demonstrate how neural embedding vectors of symbolically-encoded object affordances facilitate transferring knowledge of objects and situations to novel entities, and learning how to recognize and generate linguistic and gestural denotations.

## 1. Introduction

Over the past 15–20 years, AI has seen remarkable growth. Once beset by unmet expectations, it is now a central focus of modern computer science, with a maturing set of technologies to match (Menzies, 2003; McCarthy, 2007; Liu et al., 2018). A significant proportion of this growth has been driven by advances in natural language processing (NLP), previously a difficult problem with brittle solutions, and now a mainstay of technologies in everyday use. Developers without substantial prior knowledge of AI or linguistics can now use robust pipelines for natural language tasks such as tokenization, parsing, or speech recognition, just to name a few. Within the previous decade, the 2010s, NLP progress was kicked into overdrive, largely due to developments in deep learning and the concurrent emergence of large datasets and affordable GPUs for processing them. Deep learning has been applied to tasks such as question answering (Sultana and Badugu, 2020), dialogue systems (Zaib et al., 2020), and text generation (Iqbal and Qureshi, 2020), etc.

Many of the biggest recent successes in NLP have been driven by large, pre-trained language models, beginning with ELMo (Peters et al., 2018), and now usually based on the transformer architecture, including BERT (Devlin et al., 2018), and the GPT family (Radford et al., 2019). These language models lend themselves well to transfer learning with task-specific fine tuning, and facilitate the generation of text that is grammatical, largely coherent, and usually on-topic given an initial prompt. They are also simple to deploy and well-pipelined for general use in larger applications or just as a demonstration of the capabilities of modern NLP.

Nonetheless, despite the apparent success of language models in NLP tasks, there are a number of ways in which they fail to demonstrate “intelligence” or “understanding” as commonly defined, in particular on “tasks” that would typically be trivial for a human (Bender and Koller, 2020). In previous work (Krishnaswamy and Pustejovsky, 2019c; McNeely-White et al., 2019), we have given the example of asking a computer or smartphone the simple (for a human) question “What am I pointing at?” Put simply, current NLP systems lack the machinery to answer the question, being unable to see you or the surrounding context, and so tend to dodge the question^1^. Large predictive language models (such as multimodal BART-Large Lewis et al., 2019) appear to perform better, at least in benchmarked competitions, such as the SIMMC track at DSTC9 (Moon et al., 2020; Kottur et al., 2021). The apparent success of these models, however, is tempered when considering the nature of the task: i.e., simulated virtual multimodal shopping cart decision making. While benchmarking and evaluation are important, this is far from the fluent situated grounding we perform as humans interacting in the world every day^2^.

This is not surprising, given the nature of how such models are trained. Although trained on a enormous amount of text, these models lack knowledge of the current situational context, because that context is supplied using non-textual modalities, and so the main advertised advantage of a pre-trained language model—the ability to transfer knowledge learned from observed text to previously unencountered text—disappears. As in the SIMMC challenge, visual and multimodal transformers (e.g., Tsai et al., 2019; Dosovitskiy et al., 2020) have been trained to perform cross-modal inference on multiple tasks (Hu and Singh, 2021), but require the same or larger data sizes as unimodal transformers, and evidence suggests that accurate visual processing in a live context requires additional fine-tuning of the visual models to filter out the background (Trabelsi et al., 2021).

In the real world, we now have many usable interactive systems, such as smartphones and the entire internet-of-things, but the large datasets and compute power that facilitate high-performing NLP fail in many contexts in which we might wish to use these devices, and might expect them to function as if they truly understand us. Put simply, the current state of the technology runs up against a wall because these systems exist in a situated context (a home, an office, a car, a pocket, etc.), but lack the ability to validate information across the different modalities of description that might be implicated in all these situations. They also lack background knowledge about other entities present in the situation. Therefore, how can we expect to interface with these devices when something so basic to a human—like “What am I pointing at?”—fails?

However, the answer to this puzzle is not simply the incorporation of the right sensors into the device. Simply giving a smartphone's AI access to the camera is not enough; comparison of the literature on human object recognition vs. object recognition using computational neural networks suggests the two processes are rather different. Typical computational object recognition pipelines are usually constructed to assign a label or caption to an image. More sophisticated ones may assign heatmaps showing the region of the image being attended to in conjunction with the label or a particular word in the caption (Xu et al., 2015). While these kinds of computational vision pipelines have analogs in low-level human visual processing, low-level visual features do not fully explain how humans solve object recognition and categorize object classes (Zhang, 2010). Core recognition requires capturing properties of invariance (Riesenhuber and Poggio, 1999; DiCarlo et al., 2012), and certain computational recognition architectures have demonstrated success in using visual semantics to ground invariant representations (e.g., Garg et al., 2018). Since the 1990s, cognitive linguists have also hypothesized that semantic invariance accounts for the transfer of linguistic category labels between domains (Lakoff, 1993) while maintaining basic semantic structure.

In this paper, we will:

- Discuss our *situated grounding* approach to multimodally encoding structured context using the VoxML modeling language;
- Introduce our platform, *VoxWorld*, which demonstrates real-time modeling of context through multimodal grounding of object and event properties in a simulation environment, and the *common ground* that arises between interlocutors in the course of an interaction;
- Demonstrate the *affordance embeddings* technique, that leverages the benefits of both neural and symbolic models to address novel challenges in AI: learning the properties and description of novel objects, and learning affordance-denoting gestures.

## 2. Related work

The problem of grounding the meaning of symbols in a cognitivist approach was famously focused by Harnad (1990), who posited connectionism as a candidate mechanism for learning invariant features. In the area of human-computer interaction and dialogue, much of the foundational work in situated language understanding has its origins in the diverse areas of multimodal interface design, starting with work that combines language and gesture (Bolt, 1980), which anticipated some of the issues discussed here, including the use of deixis to disambiguate references, and also inspired a community surrounding multimodal integration (e.g., Dumas et al., 2009; Kennington et al., 2013; Turk, 2014). The psychological motivation for multimodal interfaces, as epitomized by Quek et al. (2002), holds that speech and gesture are co-expressive and processed partially independently, and therefore complement each other. Using both modalities increases human working memory and decreases cognitive load (Dumas et al., 2009), allowing people to retain more information and learn faster.

Visual information has been shown to be particularly useful in establishing common ground (Clark and Wilkes-Gibbs, 1986; Clark and Brennan, 1991; Dillenbourg and Traum, 2006; Eisenstein et al., 2008a,b), or mutual understanding that enables further communication. Many researchers in HCI have emphasized the importance of shared visual workspaces in computer-mediated communication (Fussell et al., 2000, 2004; Kraut et al., 2003; Gergle et al., 2004), highlighting the usefulness of non-verbal communication in coordination between humans (Cassell, 2000; Cassell et al., 2000).

We take the view that a “meaningful” interaction with a computer system should model certain aspects of similar interactions between two humans (Kruijff et al., 2010). Namely, it is one where each interlocutor has something “interesting" to say, and one that enables them to work together to achieve common goals and build off each other's contributions, thereby conveying the impression to the user that the computer system is experiencing the same events. Hence, the foundation of multimodal communication, be it human-human or human-computer, is based on the following criteria (Kruijff et al., 2007; Kozierok et al., 2021; Krishnaswamy and Pustejovsky, 2021).

1. Interaction has mechanisms to move the conversation forward (Asher and Gillies, 2003; Johnston, 2009).
2. System makes appropriate use of multiple modalities (Arbib and Rizzolatti, 1996; Arbib, 2008).
3. Each interlocutor can steer the course of the interaction (Hobbs and Evans, 1980).
4. Both parties can clearly reference items in the interaction based on their respective frames of reference (Ligozat, 1993; Zimmermann and Freksa, 1996; Wooldridge and Lomuscio, 1999).
5. Both parties can demonstrate knowledge of the changing situation (Ziemke and Sharkey, 2001).

It has long been clear that human reasoning is strongly sensitive to context (Stenning and Van Lambalgen, 2012; Pereira et al., 2014), and recently, earlier logical-symbolic methods of encoding context, prevalent in the AI field before the AI winter of the 1980s, have been incorporated into deep learning-driven modern AI methods as a way of including some of the structure they provide into the flexible representations provided by deep learning (e.g., Besold et al., 2017; Garcez et al., 2019; Mao et al., 2019; Marcus and Davis, 2019)^3^. The question of better incorporating contextual structure into deep learning necessarily raises the question of the analytic and structural units of context.

Context is strongly coupled to the elements of the surrounding environment in which reasoning takes place. That is, in order to conduct and describe reasoning, an agent (human or artificial) must ground its thoughts, actions, and utterances to elements of the environment (e.g., as demonstrated by Kopp and Wachsmuth, 2004). “Grounding” in much of currently-practiced NLP typically refers to kinds of multimodal *linking*, such as semantic roles to entities in an image (Yatskar et al., 2016), or joint linguistic-visual attention between a caption and an image (Li et al., 2019). Most work in the broader AI community concerned with the computational construction of reasoning environments naturally comes from the robotics community (e.g., Thrun et al., 2000; Rusu et al., 2008), or from the deep reinforcement learning (RL) community, where simulated environments are used for navigation, game-playing, and problem solving *via* deep RL (Kempka et al., 2016; Kolve et al., 2017; Savva et al., 2017, 2019; Juliani et al., 2018). These environmental platforms are not developed specifically to focus on communication, underspecification resolution, language grounding, or concept acquisition, though they may be used for these cases.

Reasoning successfully about an environment largely depends on the ability to recognize and reason about the objects that populate the environment, and a primary component of the context of objects is the actions that those objects facilitate, or their *affordances* (Gibson, 1977, 1979). An affordance is an action possibility that an item, usually an object, allows an agent. For example, chairs are can be sat on, cups can be drunk from, and handles can be grasped. Exploiting affordances can themselves give rise to other affordances, such as when grasping a door knob allows the possibility of opening the door if it is closed. Affordances in the large have been a topic of interest in many subcommunities in artificial intelligence, cognitive science, and computational language understanding (Osiurak et al., 2017). Psychological studies have shown that humans respond faster when objects are observed in canonical configurations (or *habitats*) for their typical affordances (Yoon et al., 2010). Roboticists are particularly interested in affordances, and work from that community has demonstrated that in order to successfully interact with an object, it is more important to know its function than its name. Function correlates with action and the associated hot spots of the objects enabling these affordances (Myers et al., 2015; Kulkarni et al., 2019; Allevato et al., 2020; Fang et al., 2020; Murali et al., 2020; Turpin et al., 2021). The computer vision community has also recently produced data-driven work on affordances, ranging from a focus on grasping (Tekin et al., 2019; Grady et al., 2021; Hou et al., 2021) to intention-driven human-object interaction (Xu et al., 2019).

The advent of large datasets of annotated images and video has allowed the application of many deep learning techniques toward computational processing of the objects depicted in those datasets, and their functions. Of note is work in spatial affordances for self-driving cars (Chen et al., 2015), simultaneous object and affordance prediction using deep CNN backbones (Do et al., 2018), reasoning about human-object interaction *via* dual-attention networks (Xiao et al., 2019), and predicting structural affordances such as concavity through relational graphs (Toumpa and Cohn, 2019). However, there exists a gap between many of the approaches facilitated by large datasets and the approaches to the topic as demonstrated in psychology and cognitive science: the data-driven systems are task-specific and have difficulty expanding beyond the entities they are trained over, they typically do not have a strong treatment for *habitats* (McDonald and Pustejovsky, 2013; Pustejovsky, 2013)—the configurations in which an affordance of an object may or may not be available for exploitation, and they depend on large amounts of data which makes them expensive and time-consuming to train.

One significant early attempt to model the use of language and non-verbal behavior in situated contexts is the work associated with the Collaborative Research Center's *Situated Artificial Communicator* project (Rickheit and Wachsmuth, 2006). Importantly, for our present discussion, the focus of this work was on task-oriented communicative interactions, combining language, knowledge, planning, and sensorimotor skills. The results reported in Kranstedt et al. (2008) discuss how gesture and deixis are distinguished in task-oriented communication, concerning the distinction between object-pointing and region-pointing. They further discuss the integration of deictic gesture in the determination of the semantics of a multimodal expression, through multimodal alignment. Subsequent work on how to annotate multimodal dialogue to best reflect the negotiation of common ground has resulted in annotation specifications for capturing such interactions (Tenbrink et al., 2008, 2013) as well as multimodal datasets that encode these complex interactions between gesture and language in dialogue (Lücking et al., 2013). The use of multiple modalities enrich the ways that humans and agents can communicate in situation based tasks, such as those investigated in the cognitive robotics community [e.g., Cangelosi (2010)].

The broad definitions of the goals of situated dialogue in a multimodal setting were laid out in Kruijff et al. (2007) and Kruijff et al. (2010), and have given rise to a number of fruitful and productive research avenues, as reported in Beinborn et al. (2018) and Krishnaswamy and Pustejovsky (2021). Our “situated grounding” approach uses multimodal simulated environments and exploits affordances to both facilitate learning of object properties and to compose the constraints imposed by the use of affordances to learn structural configurations (Krishnaswamy and Pustejovsky, 2019c; Krishnaswamy et al., 2019; Pustejovsky and Krishnaswamy, 2019). We have demonstrated how to exploit multimodal information to conduct learning over smaller data samples than typical end-to-end deep learning pipelines. This potential for sample efficiency suggests that situated grounding allows reusing elements of the learning pipeline to apply solutions from one task to another.

Recent work on multimodal conversational modeling (Crook et al., 2021; Kottur et al., 2021; Chiyah-Garcia et al., 2022) has pushed the boundary of what capabilities, as mentioned in Kruijff et al. (2010), can be addressed using multimodal transformer architectures, such as Chen et al. (2020) and Hu et al. (2020). There is some recent work attempting to integrate the data-driven, neurally-encoded information associated with robotic arm placement and control with linguistic symbolic guidance and instruction through dialogue (She et al., 2014; She and Chai, 2017).

## 3. Multimodal communication in context

As sophisticated as current task-based AI systems are and as intelligent as they can behave in their domains, they often fail in understanding and communicating crucial information about their situations. Robust communicative interaction between humans and computers requires that:

1. All parties must be able to recognize input and generate output within multiple modalities appropriate to the context (e.g., language, gesture, images, actions, etc.);
2. All parties must demonstrate understanding of contextual grounding and the space in which the conversation takes place (e.g., co-situated in the same space, mediated through an interface, entirely disconnected, etc.);
3. All parties must appreciate the consequences of actions taken throughout the dialogue.

Multimodal tasks rely on the contexts established between and across modalities (Matuszek, 2018), and so we propose that the difficulties faced by multimodal end-to-end systems, as well as the difficulty evaluating the state of these tasks is largely because contextual encoding still tends to be hit-or-miss, and the nature of the analytic and structural units of context, as humans use for sensitive contextual reasoning, remain the subjects of debate. This section introduces our approach to this problem: a modeling language and theoretical framework, VoxML (Pustejovsky and Krishnaswamy, 2016), that captures common object and event semantics, with a particular focus on habitats and affordances. VoxML models ontological information that is difficult to learn from corpora due to being so common that it is rarely documented and therefore not available to machine learning algorithms^4^.

Following on Clark et al. (1983); Stalnaker (2002); Asher and Gillies (2003); Kruijff et al. (2007); Tomasello and Carpenter (2007); Abbott (2008), and others, we adopt and elaborate the notion of computational common ground that emerges between interlocutors as they interact, and facilitates further communication by providing common knowledge among agents (Chai et al., 2014). Common ground is one such method of encoding and analyzing situational and conversational context (Kruijff, 2013; Pustejovsky, 2018).

We break down computational common ground into representations of:

- **A**: the agents interacting;
- **B**: the set of the agents' beliefs, desires and intentions (BDI);
- **P**: the perceived objects involved in the interaction;
- E: the minimal embedding space required to execute the activities implicated during the course of the interaction.

**Figure 1:**
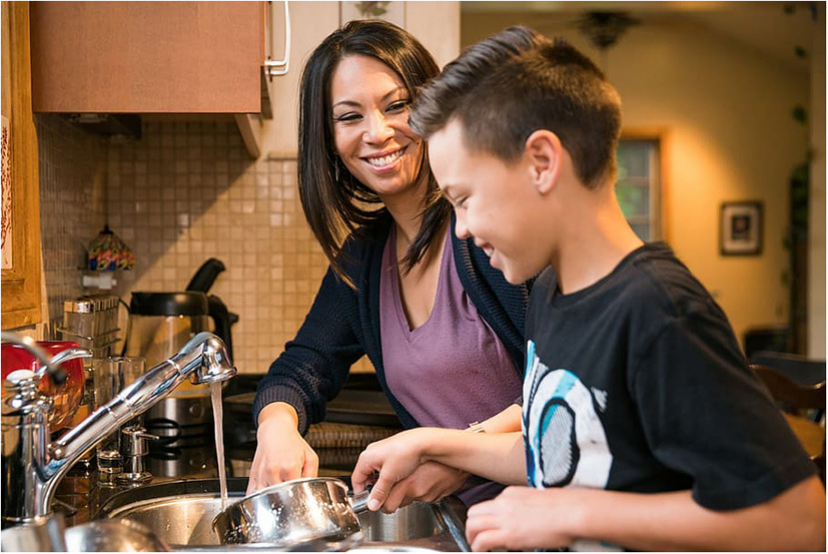
Two humans interacting in a shared task with example common ground entities.

All these parameters also include the terms used to discuss them. For instance, in Figure 1, we have a shared task involving *washing and putting away dishes*. In this context, the participants most likely agree that they share a goal to, e.g., clean the dishes, empty the sink, put the dishes away, etc. (if one of them does not share this belief, this impacts the way both of them will communicate about the task and their beliefs about it). This in turn implicates the properties of the objects involved, e.g., what it means to have a clean plate vs. a dirty plate with relation to what a plate is for.

Specific object properties are a topic of much discussion in semantics, including Generative Lexicon theory (Pustejovsky, 1995; Pustejovsky and Batiukova, 2019), and are also of interest to the robotics community (Dzifcak et al., 2009). Object properties, though important for theoretical semantics and practical applications of modern intelligent systems, pose a problem for even some of the most sophisticated task-based AI systems. A formal structure provided by the elements of common ground and situational context proposes a possible solution to these difficulties. Subsequently, we detail experiments we have been conducting in VoxWorld, the situated grounding platform based on the VoxML modeling language. These experiments combine neural learning and symbolic reasoning approaches to address transfer learning and affordance learning for an intelligent agent.

### 3.1. Modeling context

The actions facilitated, or *afforded*, by objects (Gibson, 1977) are a primary component of situational context. Gibson's initial formulation of affordances defines the term as what the environment “offers the animal.” Gibson refers to the term as “something that refers to both the environment and the animal in a way that no existing term does. It implies the complementarity of the animal and the environment” (Gibson, 1979).

We use the term in our work in a way that attempts to cover the extensive ground that Gibson uses it for, while maintaining a clear relation between the environment (including object configuration as a positioning, or *habitat*), the properties of an object that allow it to be used for certain behaviors (e.g., the “graspability” of a handle), and the language used to describe these behaviors and ground them to an environment or situation, as has been explored in recent neural AI work (e.g., Das et al., 2017; Hermann et al., 2017).

For instance, a cup standing upright on its supporting surface is in a position to be *slid* across it, while on its side, the cup is in a position to be *rolled*. Executing one or the other of these actions would require the cup to be placed in the prerequisite orientation, and may result in concomitant effects, such as anything contained in the cup spilling out (or not). These configurational constraints are encoded as *habitats* in Feature Structure (1), with the property of being upright encoded as an *intrinsic habitat* (*H*_[3]_) and being on its side encoded as an *extrinsic habitat* (*H*_[5]_).



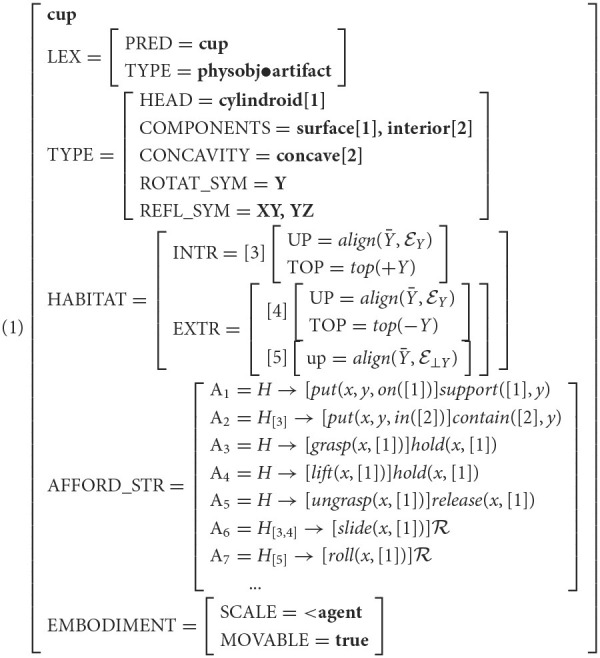



We correlate these *afforded* behaviors (a la Gibson, and Pustejovsky, 1995's *telic* roles) with the notion of habitats (McDonald and Pustejovsky, 2013; Pustejovsky, 2013), or conditioning environments that facilitate affordances. VoxML provides the format for the symbolic encodings of our neurosymbolic pipeline. Each component of a VoxML encoding, or “voxeme” (e.g., object shape, event semantic class, individual habitat, affordance, etc.) can be hand-encoded, extracted from corpora, or learned, providing a way to habituate common qualitative knowledge into a structured but flexible representation. This qualitative knowledge is important to reflect human-like qualitative reasoning capabilities in a computational context. When reasoning about a ball rolling, humans do not need to know the exact value of parameters like speed or direction of motion, but to simulate the event computationally, every variable must have a value for the program to run. VoxML provides a structured encoding of properties for these variables that allows a system to generate values when needed. Feature Structure (1) shows the VoxML encoding for a cup. Note the intrinsic upward orientation of the habitat *H*_[3]_ where the cup's Y-axis is aligned with that of the world, and the afforded behaviors that may be conditioned on a particular habitat, or may be available in any habitat (denoted *H* → ). [[cup]] has a “roll” affordance ([roll(x,[1])]R), where R simply stands for the resultant state of the process resultative (Pustejovsky and Batiukova, 2019).

#### 3.1.1. Multimodal simulations

The situated, simulated environments of the VoxWorld platform bring together three notions of simulation from computer science and cognitive science (Pustejovsky and Krishnaswamy, 2019):

1. *Computational simulation modeling*. That is, variables in a model are set and the model is run, such that the consequences of all possible computable configurations become known. Examples of such simulations include models of climate change, the tensile strength of materials, models of biological pathways, and so on. The goal is to arrive at the best model by using simulation techniques.
2. *Situated embodied simulations*, where the agent is embodied with a dynamic point-of-view or avatar in a virtual or simulated world. Such simulations are used for training humans in scenarios such as flight simulators or combat situations, and of course are used in video gaming as well. In these contexts, the virtual worlds assume an embodiment of the agent in the environment, either as a first-person restricted POV or an omniscient movable embodied perspective. The goal is to simulate an agent operating within a situation.
3. *Embodied theories of mind and mental model building*. Craik (1943) and, later, Johnson-Laird (1987) develop the notion that agents carry a mental model of external reality in their heads. Johnson-Laird and Byrne (2002) represent this model as a situational possibility, capturing what is common to different ways the situation may occur. Simulation Theory in philosophy of mind focuses on the role of “mind reading” in modeling the representations and communications of other agents (Gordon, 1986; Goldman, 1989, 2006; Heal, 1996). Simulation semantics (as adopted within cognitive linguistics and practiced by Feldman, 2010; Narayanan, 2010; Bergen, 2012; Evans, 2013) argues that language comprehension is accomplished by such mind reading operations. There is also an established body of work within psychology arguing for *mental simulations* of future or possible outcomes, as well of perceptual input (Graesser et al., 1994; Zwaan and Radvansky, 1998; Barsalou, 1999; Zwaan and Pecher, 2012). The goal is semantic interpretation of an expression by means of a simulation, which is either mental (a la Bergen and Evans) or interpreted graphs such as Petri Nets (a la Narayanan and Feldman). The aforementioned approaches cover only certain embodied theories of mind that are relevant to this work vis-à-vis the building of mental models and representations.

Bridging AI and cognitive science in this way has particular relevance to grounded natural language understanding, especially in the challenges of incorporating world knowledge, ecological semantics (Gibson, 1979; Feldman, 2006), and affordances (Gibson, 1977; Tamari et al., 2020). Krishnaswamy (2017) brings computational model testing, situated embodiment, and mental modeling machinery together into Monte-Carlo visual simulation of underspecified motion predicates, which forms the backbone of a situated approach to learning and language understanding. Given a label (symbol) of a motion verb, there may be a large space of potential specific instantiations of that motion that satisfy the label. The specifics may depend on the objects involved, and may contain many underspecified variable values (e.g., speed of motion, exact path—depending on the verb, etc.). This makes resolving underspecification ripe territory for the application of neural networks as universal function approximators.

In the *washing and putting away dishes* scenario from above, each agent maintains their own model of what the other agent knows, including respective interpretations of vocabulary items. For instance, if the mother says “pass me that plate” and the son throws it at her, it becomes clear to her that his interpretation of “pass” differs from hers. Since the computer system operationalizes all these motion predicates in terms of primitive motions like *translate* and *rotate*, it needs a model that accommodates flexible representations of these primitive motions and of their composition into more complex motions.

The Monte-Carlo simulation approach of VoxWorld provides the model in which to operationalize these complex motion predicates in ways that behave according to the preconceived notions of a typical human user. Given an input (a simple event description in English), the input is parsed and broken out into VoxML representations of the objects, events, and relations involved. These individual structured representations are then *recomposed*. From that recomposition, the variables of the composed representation that remain unassigned are extracted as the underspecified features.

**Figure 2:**
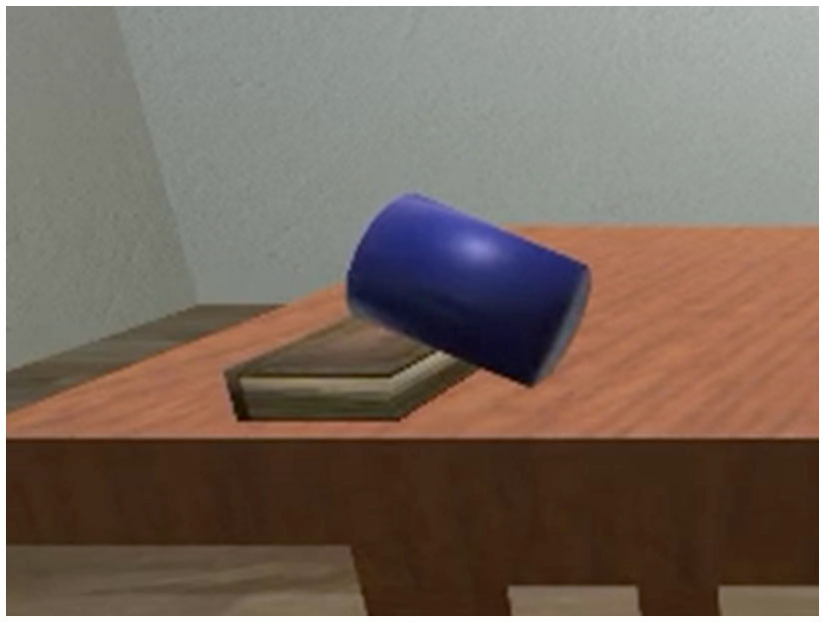
Visualization of “lean the cup on the book”.

The VoxML- and Unity-based VoxSim software (Krishnaswamy and Pustejovsky, 2016b) was then used to generate over 35,000 animated visualizations of a variety of common motion events (put, slide, lift, roll, lean, etc.) with a vocabulary of common objects (cups, pencils, plates, books, etc.), that displayed a wide variety of underspecified variables in their respective operationalizations. Every visualization was given to 8 annotators each, along with two other variant visualizations of the same input event, and the annotators were asked to choose the best one, as well as to choose the best event caption for each visualization^5^. We then extracted the range of values assigned to underspecified parameters in those visualizations which annotators judged appropriate, and used a feedforward deep neural network (DNN) to predict the best values for underspecified parameters given an event input in plain English. When given an input text, VoxSim runs the underspecified parameter symbols through the model, and the resultant output values are assigned to the relevant input parameters, resituated in the scene, and executed in real time to create an appropriate visualization of the input event. Figure 2 shows the resulting state for one such visualization for “lean the cup on the book.”

**Figure 3:**
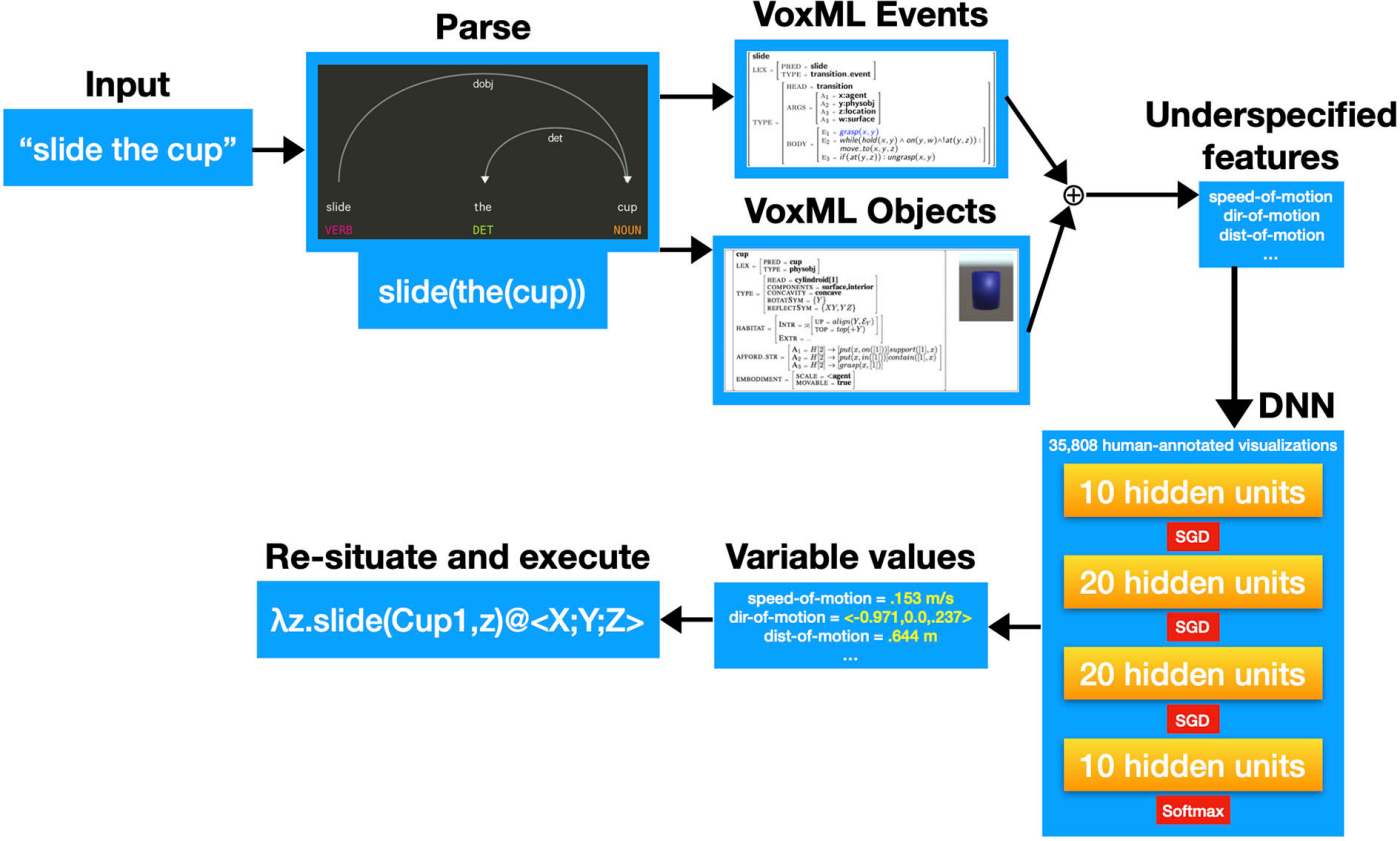
Pipeline for generating multimodal simulations.

This pipeline is shown in Figure 3 and serves as the basis for interactively exploring learning and reasoning through situated grounding and has been used to explore problems in spatial reasoning, concept acquisition for structures and novel configurations, and referring expressions (Krishnaswamy and Pustejovsky, 2019a,c; Krishnaswamy et al., 2019).

A standard VoxML library including 23 object encodings with habitats and affordances, 8 programs, and 12 relations is available with the VoxSim distribution at https://github.com/VoxML/VoxSim. Pustejovsky and Krishnaswamy (2016) contains the specification for crafting more voxemes.

## 4. Affordance embeddings for transfer learning of object properties and linguistic description

If through correlating cross-modal representations, situated grounding serves as a platform for improving sample efficiency through reuse, it should also facilitate transferring knowledge gained from solving one problem and applying it to another situation. Situatedness is particularly useful for transfer learning, because similar concepts often exist in similar situations (cf. analogical generalization, a la Forbus et al., 2017).

In Section 1, we discussed the difficulty that unimodal language understanding systems can have when answering certain simple questions pertaining to situatedness, e.g., “what am I pointing at?” In addition, top-performing object recognition and image classification algorithms of the last decade [e.g., AlexNet (Krizhevsky et al., 2012), ResNet (He et al., 2016), or Inception (Szegedy et al., 2017)] have since been shown to sometimes learn or exploit trivial or spurious correlations in the data, such as correlating object class with background features, or learning only object poses shown with little occlusion (Barbu et al., 2019). These resulting biases make it difficult for such systems to identify objects that do not occur in the large datasets they train over (e.g., a new type of chair that a human would recognize as such even if it lacks many stereotypical design features of a chair), or that occur in non-canonical framing or view (e.g., a picture of a chair taken from above).

Moreover, the reason that humans can identify novel objects as belonging to a known or similar class as well as recognize objects in unconventional situations is likely due to neural processes triggered within the brain when humans (and some primates) are presented with objects to interact with. Memories of these interactions guide subsequent analysis of other objects (Jeannerod et al., 1995; Grafton et al., 1997), making object identification in humans an inherently multimodal process. Multimodal cues allow humans to learn and “cache out” representations of objects and their associated activities into grounded semantics that can be referenced through language (Arbib, 2008; Pustejovsky, 2018).

While situated grounding provides a solution to linking linguistic terms to entities sharing the agent's co-situated space, the agent can still only discuss these entities if she knows the appropriate terms for them. If an agent encounters a new object that she doesn't know the name of, she can discuss it in terms of “this one” or “that one,” but cannot decontextualize the reference with a lexical label.

Since similar objects typically have similar habitats and affordances (e.g., cylindrical items with concavities often serve as containers), it is worth investigating whether such properties can be transferred from known objects to novel objects that are observed to have similar associated properties.

The method we use is termed *affordance embedding*. This follows an intuition similar to the Skip-Gram model in natural language processing (Mikolov et al., 2013), or the masked language model of BERT (Devlin et al., 2018), but exploits the linkage between affordances and objects present in a modeling language like VoxML, which is tightly coupled to simulated physics of the real world.

As an example, [[cup]], as shown in Feature Structure (1), only affords rolling under a particular condition, encoded as *H*_[5]_, which requires that the cup be placed with its Y-axis (Ȳ), the same axis of symmetry it shares with [[ball]], perpendicular (⊥) to the Y-axis of the embedding space E. Compare this to a ball, which is rollable no matter its orientation. Therefore, [[cup]] is similar to [[ball]] in terms of its rollability in general, but may not be *most* similar in terms of the circumstances under which rolling can occur, and so may not be as similar in terms of other affordances such as grasping or containing. What is novel about our approach here is that by using affordances to learn correlations to other affordances without learning the object label directly, we explicitly target the problem of handling objects not encountered in the training vocabulary (see Sections 4.3, 4.4), we consider the effect of both habitats and affordances on object reasoning, and we demonstrate our method's performance on the task even though it is only trained over a small sample size.

For affordances to be truly useful in learning about new concepts, they need to demonstrate accuracy in analogizing new entities to existing ones and the ability to make such predictions from a small amount of prior data. Here we use habitats and affordance information derived from a purposely small dataset to address object similarity in a situated grounding context. In the remainder of this section, we will:

- Detail our methods for analogizing objects from their habitats and affordances;
- Present results showing the accuracy we can achieve using different variations on these methods;
- Show how we deploy the resulting models in real-time interaction;
- Demonstrate how to correlate newly-learned gestures to object grasping actions.

### 4.1. Methodology

To automatically explore affordances such as *grasping*, a system must have an agent capable of grasping items, namely an *embodied, situated agent* that explores its situation and grounds its reasoning to its own dynamic point of view. In Krishnaswamy et al. (2017) and Narayana et al. (2018), we examined the problem of situatedness and communication within a situated context in an encounter between two “people”: an avatar modeling multimodal dialogue with a human.

**Figure 4:**
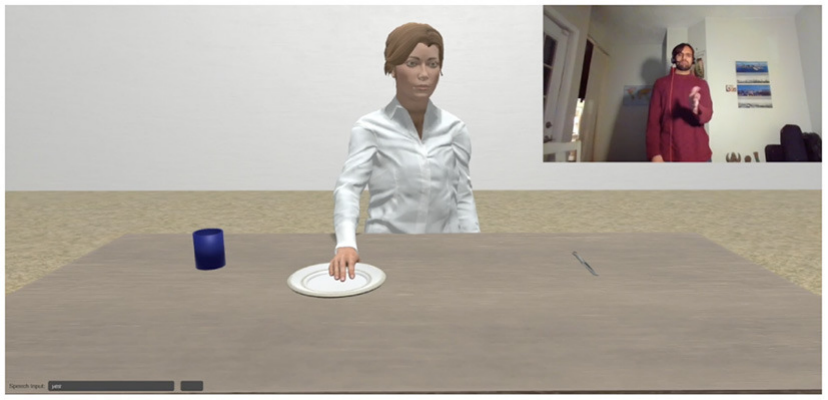
Diana interacting with a human in the “Kitchen World” environment.

Our agent in VoxWorld, known as Diana, is situated in a virtual VoxSim environment (Figure 4). A human interlocutor can give Diana instructions about what to do with objects in her virtual world using both spoken language and live gesture, making Diana an interactive collaborator^6^.

Here we will discuss a zero-shot transfer learning method using objects available to the Diana agent. Our dataset, code, and results may be found at the link below^7^.

#### 4.1.1. Training data

The data we use comes in the form of VoxML-encoded objects that drive the semantic processing in the Diana system (e.g., McNeely-White et al., 2019). These datasets typically contain separate encodings for distinct objects of the same type, e.g., “red block” vs. “blue block” or other type-token distinctions including those based on attributive qualities, so we strip the data down to contain one VoxML encoding per voxeme type. This leaves us with 17 distinct object types to train on: *apple, ball, banana, blackboard, block, book, bottle, bowl, cork, cup, disc, grape, knife, paper sheet, pencil, spoon*, and *table*.

These objects contain a diverse set of habitats describing configurational and size constraints and a diverse set of affordances including many behaviors in common, such as *grasping*, and many distinct behaviors that are conditioned on particular circumstances, including *supporting, containing, rolling*, and *sliding*.

Given an affordance structure taken from a voxeme (compare to Equation 1 above), there will be, for each affordance, an encoding *H*_[*N*]_ → [*E*]*R*, where *H*_[*N*]_ refers to the habitat encoding and points to an enumerated element of the intrinsic or extrinsic habitat structure^8^, [*E*] refers to the event that can be executed if the object is conditioned by habitat *H*_[*N*]_, and *R* refers to the result. For example, in the affordance *H*_[3]_ → [*put*(*x, y, in*([2])]*contain*([2], *y*) from the [[*cup*]] object:

- *H*_[3]_ points to the intrinsic “upright” habitat of the cup, being the typical orientation;
- In this habitat, if some agent *x* puts object *y* *in* subcomponent [2] of the cup (that being the interior),
- then the cup *contain*s that object.

To train our affordance embedding models, we create a word2vec-style Skip-Gram-style algorithm using Keras. The inputs to this algorithm are ASCII representations of the affordance encodings as discussed above. We split the encoding on → , leaving a tuple (*H*_[*N*]_, [*E*]*R*). Because the *N* in *H*_[*N*]_ may be ambiguous across objects (e.g., the habitat denoted *H*_[1]_ in two respective objects may be different), *H*_[*N*]_ then looks up the equivalent habitat in the voxeme's habitat structure and replaces *H*_[*N*]_ with that. Thus, if the same habitat (e.g., “upward”: [up = $align\left(Ȳ,E_{Y}\right)$, top = *top*(+*Y*)]) is denoted as *H*_[2]_ in object voxeme but *H*_[3]_ in another, they will be normalized and be vectorized the same way.

#### 4.1.2. Learning model

Affordance embeddings are designed to exploit the correlations between habitats and affordances over a vocabulary of known objects, and to map those correspondences to novel objects that are observed to possess some known habitats or affordances. We then infer in what manner those affordances may be enacted on the novel object, by comparing it to a known object. For instance, a habitat-affordance pair for a [[cup]] voxeme might be (*H*_[3]_ = [up = $align\left(Ȳ,E_{Y}\right)$, top = *top*(+*Y*)], *H*_[3]_ → [*put*(*x, in*(*this*))]*contain*(*this, x*)) (gloss: *the cup's Y-axis is aligned upward with the Y-axis of the embedding space, and if something is put inside the cup, the cup contains that thing*). The model outputs probabilities for each individual habitat or affordance. Subsequently, for each possible action the agent may take with an object (e.g., grasp, pick up, move, slide, put on, etc.), the system queries the learned affordance embeddings, *excluding* those affordances that include the particular action in question. Conceptually, this restates the answer to a query, e.g., “describe the appropriate habitat for *grasping* an object” in terms of *other* actions that can be taken in that habitat, and the habitat is matched to other objects that share that habitat. This is effectively a second-order collocation. Other objects that share a similar habitat are likely to share a similar affordance, and perhaps also other affordances that do not depend on the habitat in question, allowing us to potentially learn how to, say, *grasp* a new object from inferring something about the *containment* properties of another object.

Because the data is sparse due to the small sample, we elected to use a Skip-Gram style model (although İrsoy et al., 2021 suggests that a corrected CBOW model can perform as well as Skip-Gram on sparse tasks). We provide one affordance as the “focus word" and optimize the model as it attempts to predict additional associated affordances as the “context words.” We use both habitats alone as tokens when training the embeddings, and also affordances along with their conditioning habitats as additional tokens, so that the model trains to optimize for predicting coocurring habitats and coocurring affordances simultaneously. Because habitats are conditioning environments on affordances, habitats may occur alone but affordances do not occur without conditioning habitats. The result of this process is a model that is optimized to predict habitats and affordances based on other habitats and affordances they cooccur with. Therefore, under this assumption an object can be represented as a collection of afforded behaviors, each of which was originally symbolically encoded but is vectorized for semantic computation.

Our pipeline is implemented in TensorFlow using the Keras API. We begin by training 200-dimensional habitat and affordance embeddings using the previously discussed Skip-Gram model. This model is trained for 50,000 epochs with a window size of 3. The resultant embeddings serve as important input features to the object prediction models.

We then represent an individual object in terms of its habitats or affordances. This involves reducing the structured VoxML encoding to a single vector with minimal information loss. Fortunately, affordance encodings in VoxML encode dependencies on habitats without including dependencies on other affordances [that is, VoxML convention is to encode resultant states of affordances as distinct habitats that themselves facilitate other behaviors (Pustejovsky and Krishnaswamy, 2016)], so given an affordance structure that has largely conditionally independent components, we can represent the object as an *average* of the individual habitat and affordance vectors. When testing, out-of-vocabulary habitats or affordances are not included in the average because they add nothing informative to the model for this task; when analogizing a novel object to known objects the vast majority of information comes from what the model can already infer about the known object. These averaged embeddings form the object representations that the prediction models are trained over.

We used two separate architectures to learn object prediction from affordance embeddings: a 7-layer feedforward multilayer perceptron (MLP) as an initial proof-of-concept and a somewhat more sophisticated 4-layer 1D convolutional neural net (CNN), due to 1D CNNs' demonstrated utility in human activity recognition (Cruciani et al., 2020) and text classification (Gargiulo et al., 2018). A summary of both model architectures are give in Table 1.

**Table 1:** MLP (L) and CNN (R) architectures.

**MLP**	**CNN**
Input	Input
**Dense (32** × *tanh***)**	**Conv1D (64** × *ReLU***)**
20% Dropout	*ReLU*
**Dense (196** × *ReLU***)**	20% Dropout
20% Dropout	**Conv1D (250** × *ReLU***)**
**Dense (92** × *tanh***)**	Global Max Pooling 1D
20% Dropout	20% Dropout
**Dense (196** × *tanh***)**	**Dense (196** × *tanh***)**
**Dense (92** × *ReLU***)**	20% Dropout
**Dense (32** × *tanh***)**	*ReLU*
**Output (** *softmax* **)**	**Output (** *softmax* **)**
70,913 params	100,923 params

Bolded lines indicate layer types.

All models were trained for 1,000 epochs with a batch size of 100. We performed 17-fold cross-validation on each of these architectures, holding out each one of the objects in turn. We train each kind of architecture on habitats alone and on habitats and affordances together, for the reasons discussed above. Hereafter an “affordance-based” model refers to one trained by including habitat-affordance coocurrences in the inputs to the embedding model.

A classifier trained on all objects but “bottle” will predict the most similar object to a bottle based on the observed habitats and affordances of the bottle. Given an input object with an affordance like “grasping,” it should predict an object that a bottle can be grasped similarly to.

#### 4.1.3. Ground truth

We must assess the results of the prediction model against an established ground truth. This presents a problem as equivalent human judgments are qualitative (i.e., one person may judge a bowl most similar to a cup while another might judge it most similar to a plate, while a third adjudicator might consider both comparisons equally valid). Therefore, we presented a set of 7 annotators, all adult English speakers with at least some college education, with the object set in use in the training data, and asked them to list “*[f]or each object, which 2 other objects in the list are most similar to it, in terms of shape/structure and the things you can do with it*.” They were given no other information, no briefing on the affordance embedding task, and no access to the VoxML encodings for the objects.

We computed a Fleiss' kappa score (Fleiss and Cohen, 1973) of approximately 0.5520 over the annotations to assess the level of inter-annotator agreement (IAA), with a standard error of 0.0067 (with an assumption that the null hypothesis is that κ = 0). According to Fleiss and Cohen's informal metrics, this constitutes “moderate” agreement, but the annotation was also made more complex due to the fact that annotators were asked to make *two* choices per object rather than one, and there were many cases where annotators agreed on one object-object similarity while disagreeing on another. Some downstream effects of this are discussed in Section 4.2.

The annotation gave us 119 non-distinct object triplets, e.g., {*apple*, *ball*, *grape*} which we then plotted in 3D space according to the object indices in the vocabulary, and used to conduct k-means clustering to provide us with an automatically quantifiable *proxy* for ground truth against which to assess the object prediction. Human annotations provided the initial raw data that was converted into clusters in 3D space to quantitatively assess the performance of the model. These clusters are what the model is assessed against.

We conducted clustering using *k* = 6; for a test set of 17 objects where annotators were asked to group them into non-exclusive sets of 3, 6 means most closely approaches an average cluster size of 3 objects. Subsequent prediction results were considered a “true” positive if the predicted object (e.g., [[cup]]) clusters with the ground truth object (e.g., [[bowl]]). We assess two metrics: the percentage of results in which the prediction correctly clusters with the ground truth across the five trials in that iteration, and the percentage of time the prediction *always* clusters with the *modal*, or most commonly occurring, cluster containing the ground truth object.

### 4.2. Results

We assess two types of baselines for comparison. First, we test pretrained GloVe embeddings (Pennington et al., 2014) and the word2vec Skip-Gram model, two well-established word embedding methods, on their ability to determine vector similarity between the lexemes for objects in our vocabulary. This serves as an assessment of object similarity determination based on linguistic data alone, without access to any multimodal information, such as affordances. Second, we use the VoxML encodings directly to establish a heuristic baseline by assessing object similarities based on Jaccard distance (Jaccard, 1912) between their respective habitats and affordances. The Jaccard distance calculation is simply the intersection over union of affordances of the test object with each candidate object, and we choose the top 5 candidates based on this method. Results of these baselines were assessed relative to the ground truth clusters established in Section 4.1.3.

Using our own models, we ran a total of 340 individual trials: 5 tests with each of the 17 hold-out objects evaluated against the clusters derived from the human annotation, run by each architecture trained over habitats or affordances. Table 2 shows the accuracy results for baselines and each model-data pair.

**Table 2:** Prediction accuracy results with 6 means.

	**% predictions**	**% predictions always**
**Model**	**in correct cluster**	**in correct cluster**
GloVe embeddings	50.20	13.18
word2vec embeddings	48.37	13.23
Jaccard distance	66.67	19.28
MLP (Habitats)	78.82	27.06
MLP (Affordances)	**84.71**	38.82
CNN (Habitats)	78.82	27.06
CNN (Affordances)	81.18	**40.00**

Bolded numbers indicate best performing model for metric.

Our models perform broadly similarly, achieving upward of 75% prediction accuracy relative to the ground-truth clusters in all cases, exceeding 80% accuracy in some cases, and consistently exceeding the performance of the unimodal and purely heuristic baselines by upward of 10–20%. We believe this is because the affordance embedding models capture dependencies between an object's encoded structural constraints and its behavior in a way that is not captured by the linguistic cooccurrence captured by pretrained static word embeddings (cf. the example in Section 3.1 referencing the BERT vectors for “ball” and “round”), or by the simple intersection-over-union approach of Jaccard distance. Instead, we use the symbolic encodings to construct neural representations and use those to make similarity-based predictions of symbolic class labels. Thus, the percentage of times when the predictions *always* cluster correctly with the ground truth across all five individual trials in the same conditions is lower but still well in excess of a random chance baseline of $\left[{\left(\frac{1}{k}\right)}^{5}\times 100\right]$% and always above $\left(\frac{1}{k}\times 100\right)$%.

We believe this shows that even with a very small dataset, habitat and affordance information is very informative and useful when predicting similar objects and can function effectively with sparse data using a small and efficient model.

We see some artifacts of the clustering that arise from the annotator judgments. For instance, some annotators grouped *apple* with *grape* and *ball*, presumably due to their round shape, which captures the *roll* affordance of all those objects. However, other annotators grouped *apple* with *grape* and *banana* due to all being types of edible fruit, even though *eat* was not an affordance in the vocabulary used. Therefore, one cluster that arose frequently was {*apple*, *ball*, *grape*, *banana*}, even though annotators that grouped *apple* with *ball* tended to group *banana* with *bottle, pencil*, or *knife* (reflecting similar shapes). Artifacts like these tended to negatively affect the assessment for objects predicted to be similar to objects such as *banana*, and reflect a need for more rigorous assessment of the qualitative ground truth proxy.

#### 4.2.1. Statistical analysis

We surmise that if habitat and affordance encodings were not informative features in predicting object similarity, then classifiers trained on affordance embeddings would not consistently predict the same objects for a given set of input affordances; the null hypothesis here is therefore that affordance embedding-trained classifiers would perform no better than noise.

In evaluating the significance of the results, we treated every affordance-based classifier as an “annotator” and computed a Fleiss' kappa value following an IAA calculation similar to that which we performed over the ground truth cluster annotations (Section 4.1.3).

We use standard statistical techniques for identifying outliers, such as *z*-score filtering and normalization (Rousseeuw and Hubert, 2011). Because a single outlier can make the standard deviation large, it is common to use the median of all absolute deviations from the median (MAD) as a more robust measure of the scale (Leys et al., 2013).

When outliers, defined as when a classifier trial makes a judgment that does not concur with the judgment of any other trial of any classifier (i.e., singletons), are included, κ≈0.3006 with a standard error of approximately 0.0216, but when singleton outliers are excluded, κ≈0.7139 with a standard error of 0.0731. These values are then used to calculate a *z*-score:


$$\begin{array}{l} z=0.3006/0.0216\approx 13.8995\text{(outliers included)}\\ z=0.7139/0.0731\approx 9.7643\text{(outliers excluded)} \end{array}$$

Converting this to a *p*-value yields *p* < 0.001 in both cases. The kappa value measures *agreement* rather than *correctness*, and so it, and the accompanying z-score and *p*-value should be viewed in conjunction with the accuracy of the affordance embedding classifiers shown in Table 2.

### 4.3. Discussion

It is less useful to assess how affordance embedding-based models perform in the abstract over a diverse object set when we are more concerned with predicting similarities with *particular* novel objects as might be encountered by an agent *in situ*. We therefore kept objects out of the dataset entirely, such that they were not used in any training or cross-validation, but contained similar habitats and affordances to objects in the dataset.



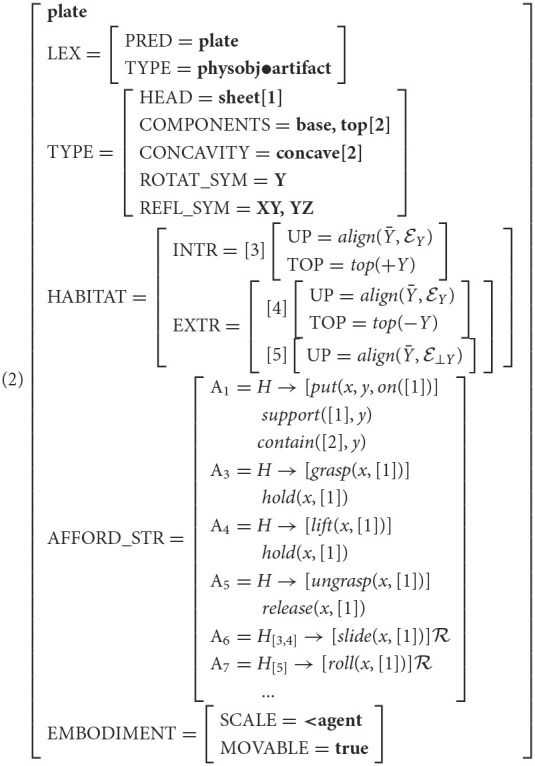



One such object is *plate*. Feature Structure (2) shows the VoxML structure for [[plate]]. Compare this with [[cup]], as shown in Feature Structure (1), to see similarities in habitats and affordances. A container like *cup* or *bowl, plate* also shares *support* affordances with, e.g., *block* and *table*, and is rollable like non-container objects like *apple*. Over 10 tests against *plate*, the baselines and classifiers each produced the following most frequently-predicted objects (in descending order of frequency):

- **GloVe embeddings**: ball, table, bowl, bottle, knife
- **word2vec embeddings**: spoon, ball, knife, table, disc
- **Jaccard distance**: bottle, cup, bowl, cork, knife
- **MLP (Habitats)**: book, cup, bowl, bottle
- **MLP (Affordances)**: cup, bottle, apple
- **CNN (Habitats)**: book
- **CNN (Affordances)**: cup, bottle

The affordance embedding models predict commonalities with other containers, rollable objects that have similar habitat constraints, and objects that have similar grasps.

From these results on an individual object, we can begin to speculate about some of the features that each model is capturing. First, we observe that the baselines do not perform particularly well in a qualitative analysis in this test, either. The pretrained GloVe and word2vec embeddings seem to capture common cooccurrence context between plates and other common tabletop items in the vocabulary, but there appears to be little systematic correlation between the typical uses of these objects. In fact, we hypothesize that the correlation in the model between “plate” and “ball” might actually be influenced by the cooccurrence of these terms in the context of baseball! The Jaccard distance metric performs slightly better, operating directly over the habitats and affordances, but still predicts one object that has only a *grasp* affordance in common with plate: a knife—and that grasp behavior is rather different in terms of hand pose.

**Figure 5:**
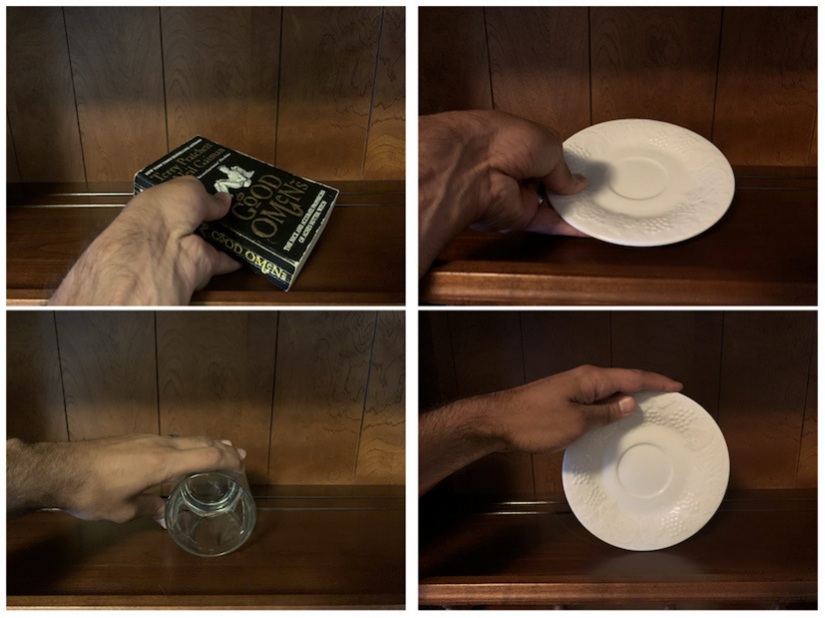
**Top:** Grasping a book vs. grasping a plate. **Bottom:** Rolling a cup vs. rolling a plate. The “rolling” habitat places the objects in the same orientation, e.g., with the cup's opening parallel to the ground.

Meanwhile, when trained solely on habitat embeddings, both the MLP and CNN models, while capturing containers similar to *plate*, also tend to predict *book* as the most similar object. The CNN model in particular predicted only *book* as similar to plate. We surmise that habitat embeddings, being sparser overall, tend to predict correlations between behaviors that are common over very many objects, such as *grasping*, and that *book* and *plate*, having similar dimensional constraints, are predicted to be grasped similarly (see Figure 5, top).

The models trained on affordance embeddings as well appear to be better at capturing more specific behavioral affordances, commonly predicting similarity between containers like *cup* and *bottle* and a plate, which might also be rolled in the same way (see Figure 5, bottom, where a non-container like an apple would be rolled in the same way). Cups and bottles are not grasped like plates, suggesting that the affordance-based model is discriminating between common behaviors like grasping that are available in almost any habitat and more object- and habitat-specific behaviors.

These results show that objects can be analogized to each other in terms of their behaviors, and these analogies can be made more specific and accurate by comparing both the afforded behaviors and the habitats in which they occur. That is, if an agent encounters an object for which she has no name but can determine that it has a number of affordances in common with another object, she can use that second object as a starting point to reason about the first.

### 4.4. Deployment

The situated grounding mechanisms provided by an embodied agent like Diana and the models learned from affordance embeddings allow the agent to discuss, learn about, and manipulate novel items that she comes across in her virtual world, including the objects in our domain.

Having established that similar objects share similar affordances; developed a method for selecting similar objects based on their configurations, constraints, and behaviors; and provided a way for the agent to estimate grasp poses in real time, we can now ask the question: what happens if the agent encounters an unknown object in her virtual world?

The affordance embedding model runs in a Python client connected *via* socket to the rest of the Diana system running in Unity. The avatar sends a behavior (“grasp”) and the set of affordances of the novel object. The model returns an object that satisfies that behavior using similar affordances.

**Figure 6:**
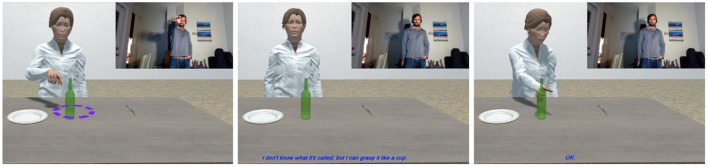
(1) Human: What is that?; (2) Diana: I don't know, but I can grasp it like a cup; (3) Human: Grab it. + resultant grasp.

For example, if the agent comes across an unfamiliar object that appears to share the *H*_[2]_ = [up = $align\left(Ȳ,E_{Y}\right)$, top = *top*(+*Y*)] (upward alignment) habitat of [[cup]], she can infer that it might be grasped similarly. Figure 6 shows this process enacted through interactive multimodal dialogue. In frame 1, the human points to a new object (recognizable as a bottle, but the agent has no label associated with it). In frame 2, the agent says “I don't know”—reflecting the semantic gap in her vocabulary—“but I can grasp it like a cup”—reflecting the information about it that she is able to infer from its habitats and affordances, which gives her a way to talk about this object with her human partner. In frame 3, the human says “grab it,” and the agent demonstrates her inferred method of grasping, based on the object similarity predicted from affordance embeddings. The way the hand is positioned is described later in this paper, in Section 4.5.

Because we use pre-existing VoxML encodings generated for a specific purpose, our training data is necessarily small. However, our results suggest that we can use transfer learning *via* affordance embeddings to generate partial knowledge about novel objects, as we demonstrate with overall accuracy in Section 4.2, and with the specific examples of plates and bottles in Section 4.3 and here above.

We have been able to demonstrate that a fairly simple affordance embedding technique inspired by Skip-Gram can yield promising results, which suggests that knowledge of habitats and affordances provides a significant amount of information per sample toward classifying action-based object similarity in a way that the cognitive science literature suggests humans do as well. We demonstrate effectiveness at predicting similar objects based on their structure, configuration, and attached behaviors using simple, straightforward architectures, and much less data than attempting to learn the same correlations from unstructured text.

### 4.5. Interactive learning of object grasping

Section 3.1.1 discussed how parameters in a predicate may be underspecified, and how Monte-Carlo sampling can be an effective way of determining the distribution of values that satisfy the predicate. However, underspecified parameters in a predicate can also be inferred from the properties of objects, namely the habitats which they can occupy and the behaviors afforded by them. For instance, if a cup is both *concave* and symmetric around the *Y-axis*, then there is no need to explicitly specify the orientation of the concavity; we can infer that it is aligned with the object's Y-axis, and this in turn requires that certain conditions (habitats) be enforced for certain affordances to be taken advantage of, such as putting something in the cup, or grasping the cup appropriately in order to drink from it (Krishnaswamy and Pustejovsky, 2016a).

Diana consumes input from 3rd party or custom speech recognition, and can see her human interlocutor's gestures with custom recognition algorithms running on deep convolutional neural networks trained on over 8 h of annotated video and depth data from a Microsoft Kinect^TM^.

One of Diana's default vocabulary of 34 gestures is a downward-opening “claw” gesture used to mean *grasp*. This gesture is sufficient to signal how to grasp an object such as a block. However, in Diana's “Kitchen World” scenario, containing common household objects including those used in the affordance embeddings training pipeline, she comes across items, like plates or bottles, that cannot be grasped in this way. In that case, she must estimate positions on the object where it is graspable.

*Grasp-point inference* uses the symmetry of objects as encoded in VoxML. Objects have rotational and reflectional symmetry, such that a cup has rotational symmetry around its Y-axis and reflectional symmetry across its XY- and YZ-planes, while a knife has only reflectional symmetry across its YZ-plane in default orientation.

**Figure 7:**
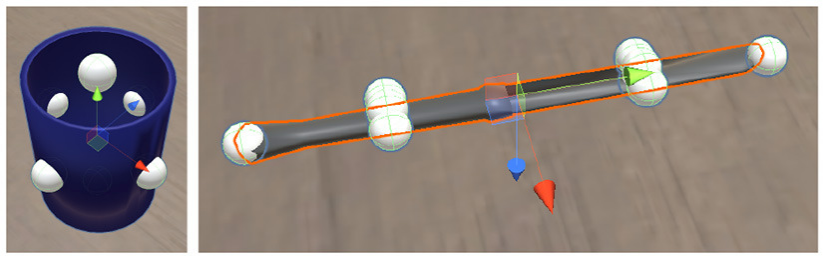
Grasp points (white spheres) on a cup and knife.

For objects with rotational symmetry, we find all points *P* on the surface equidistant from the extremes along the axis of symmetry, as well as the extreme points of the object along that axis. For objects without rotational symmetry, we find those points *P* on each component of the object that intersect the plane(s) perpendicular to the plane of reflectional symmetry (see Figure 7). The closest one of these points to the position of the agent's hand (*w*) is taken to be the targeted point of interaction with the object.

From this point we calculate the maximum amount each finger (*f*) can bend toward the object without intersecting its bounds, take this distance of the fingers to wrist joint and add it to/subtract it from the object's extremities in both directions along all 3D major axes (*extents*_*x, y, z*_):


$$\begin{array}{l} \left(3\right)\;\forall f\in F\underset{m∠fwp}{\mathrm{argmin}}|\vec{fw}|\times cos\left(m∠fwp\right)\pm extents_{x,y,z} \end{array}$$

The angle of the fingers at this point becomes a *grasp pose*, or hand position needed to activate the *grasping* affordance (that is, to exploit the *graspability*) of that object at that position. Each possible grasp pose comes with an associated affordance semantics of the form λ*y*.*grasp*[*obj, with*(*y*)] where *y* represents the associated iconic gesture. This is the symbolic portion of the new gesture semantics, which must then be filled with the actual gesture.

#### 4.5.1. Assigning affordance-denoting gesture

A portion of Diana's interaction is written such that it can be used to trigger a one-shot learning process that can add a new gesture to her existing vocabulary and assign a specific action to it. In this case, the action in question is grasping a specific kind of object.

During interaction, Diana demonstrates each inferred possible grasp pose to the human and asks for a preference. The bolded text indicates the portion of the interaction that activates the learning pipeline (Dialogue 1):

Dialogue 1 **Agent eliciting an affordance-denoting gesture.**

HUMAN: The plate.

DIANA: Okay. [ DIANA *points to the plate.* ]

[HUMAN *makes “claw down” gesture.* ]

DIANA: Should I grasp it like this? [ DIANA *grasps plate from beneath.* ]

[ HUMAN *makes “thumbs down” gesture.* ]

DIANA: How about like this? [ DIANA *grasps plate from side.* ]

HUMAN: Yes.

DIANA: **Is there a gesture for that?**

[ HUMAN *makes “grasp plate” gesture.* ]

**Figure 8:**
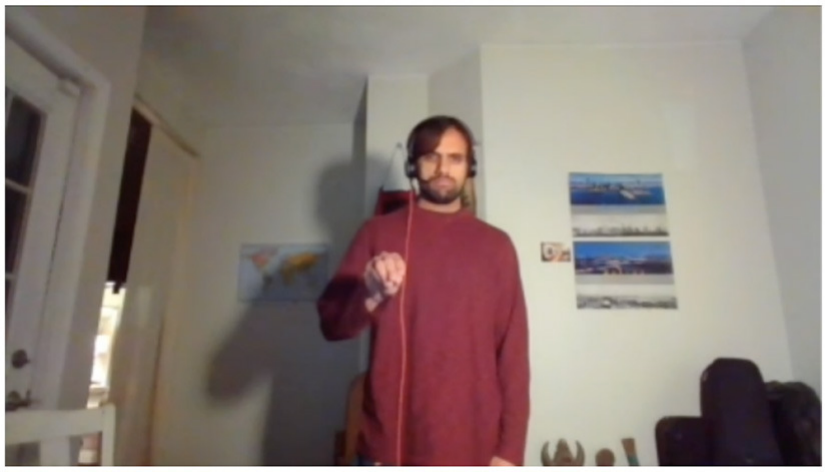
Iconic *plate* gesture for “grasp the plate”.

Now, with the affordance semantics available to be filled, the visual features of the novel gesture the human makes are fed into a random forest classifier trained over 2,048-dimensional feature vectors derived from the annotated video data used to train the default gesture recognizer. The novel gesture is situated in the feature space of the 34 known gestures (plus any novel gestures previously learned). That new vector value is applied to the outstanding variable in the affordance semantics generated through the interaction to this point. The result represents an operationalization of *grasp*(*x*) where *x* is the object requiring novel exploitation of its affordances to grasp it. This operationalized predicate is then propagated down to any other events that use [[grasp]] as a subevent over the object *x*. This now allows the human to instruct the agent to grasp an object using the correct pose, with a single visual cue, as in Figure 8. Furthermore, the avatar can subsequently be instructed to perform any actions that subsume grasping that object.

**Figure 9:**
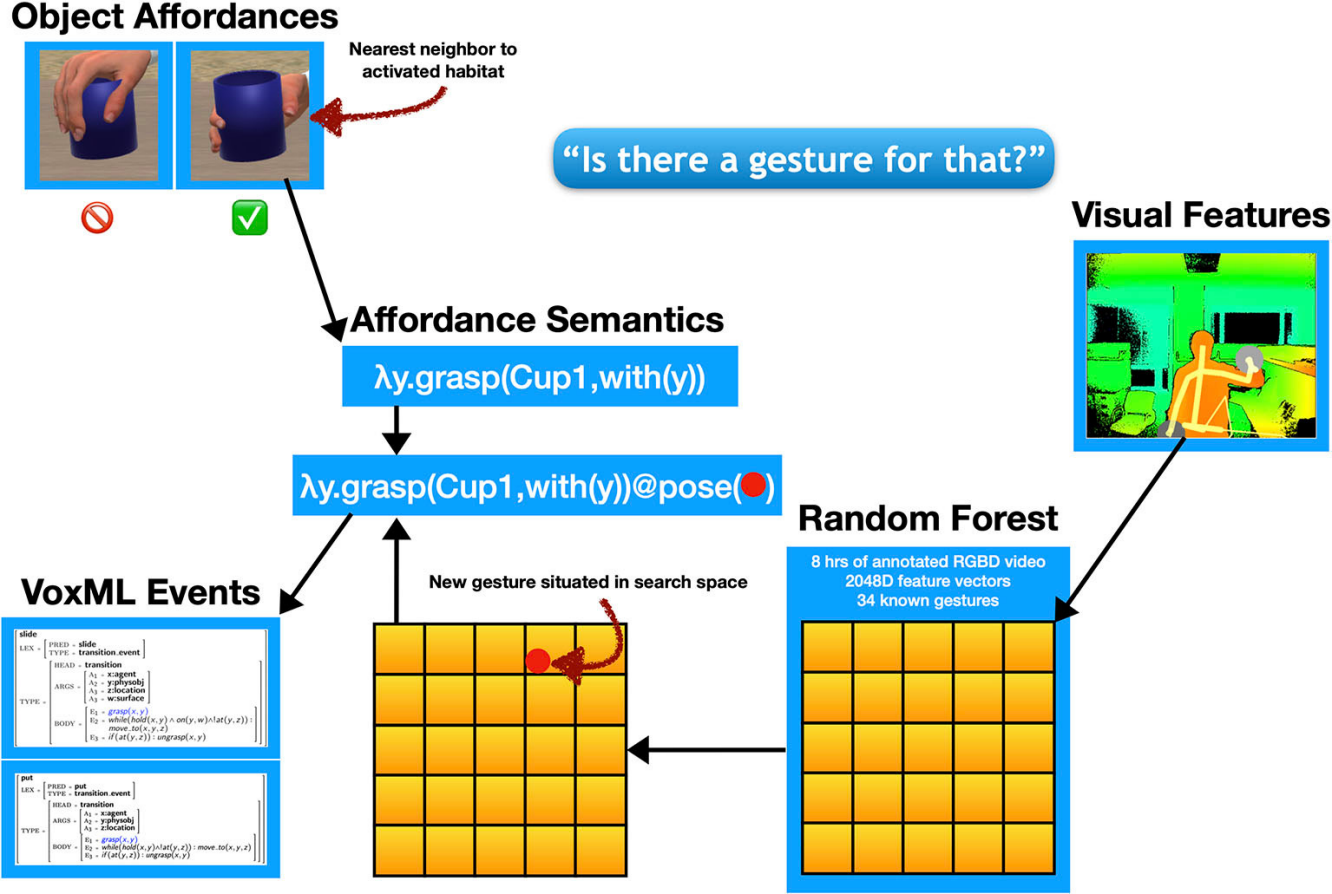
Neurosymbolic pipeline for learning interactions with object affordances. The new visual features are situated in the feature space of a deep convolutional neural model and assigned a new label.

Figure 9 gives the neurosymbolic learning pipeline for object affordances and accompanying actions. This pipeline is activated when Diana says “*Is there a gesture for that?*” Because the learned object affordance is propagated down to other events that contain the associated action, we can fill in other action sequences with this association using a continuation-passing style semantics a la Van Eijck and Unger (2010); Krishnaswamy and Pustejovsky (2019b). For example, extending the dynamic event structure developed in Pustejovsky and Moszkowicz (2011), the VoxML encoding of the event [[slide_to]] can be represented as in (4). This is a derived event composed from the activity [[slide]] and the directional PP [[to_loc]] (Pustejovsky and Krishnaswamy, 2014).


$$\begin{array}{l} (4)\;grasp(e_{1},\text{AG},y);\;while[hold(\text{AG},y)\wedge on(y,\text{SURF})\wedge \\ \;\neg at(y,\text{LOC})],move\_to[e_{2},\text{AG},y,\text{LOC}]);\\ \;if[at(y,\text{LOC}),ungrasp(e_{3},\text{AG},y)] \end{array}$$

Therefore, if the agent encounters a [[slide]] action with an outstanding variable [λ*y*.*slide*(*y, loc*)], and the human supplies a gesture denoting *grasp*(*plate*), then the agent can directly lift *grasp*(*plate*) to the slide action and apply the argument *plate* to *y*: λ*y*.*slide*(*y, loc*)@*plate*⇒ *slide*(*plate, loc*). **while**(*C, A*) states that an activity, *A*, is performed only if a constraint, *C*, is satisfied at the same moment. Here, should something cause the agent to drop the object or, should the agent lift the object off the surface, the constraint, and therefore the overall [[slide]] action will cease and remain incomplete.

Once a gesture has been learned for a particular object, e.g., [[cup]], the same gesture can be reused to signal the agent to grasp not only instances of that same object, but also objects that agent has inferred can be grasped similarly, as through the affordance embeddings transfer learning method. What this does is then connect the affordance-based inference, wherein Diana can *speak* about an object in terms of what it is similar to, even if she doesn't know what it is called, to an ability to respond to and generate pertinent gestures about it, giving her a handle on inferring partial information about the new object *via* two modalities at play in situated communication: gesture and language.

## 5. Discussion: Data and representation

In this research we have deliberately eschewed relying on the large training datasets in use by much of the AI community. This was a conscious choice: nevertheless, the question of the trade-off between the methods proposed above, requiring rich prior knowledge, and automatic acquisition of implicit information through larger-scale machine learning, needs to be addressed.

In this we believe it is important to consider how the history of AI has progressed to this point. Early AI saw great promise in symbolic representations, presenting computers as powerful symbol-processing machines, but these hopes were not borne out, leading to the infamous AI winter. One early proponent of AI approaches decoupled from explicit representation was Rodney Brooks (1991) who notably viewed the shortcomings of symbolic AI as including its lack of capturing either situatedness or embodiment with regard to robotics. Brooks was not a core researcher in neural networks. Nonetheless, since the mid-1990s, neural network approaches, once disregarded, have grown to dominate the field through their ability to uncover patterns implicit in data and apply those patterns to various different tasks. The notion of extracting relevant information from the data, or “world” (a la Brooks) itself has proven to be very powerful.

Nevertheless, when we take a closer look at where the training data for the most successful models comes from, it becomes clear that the successes of large models comes not just from the ability to find patterns in data, but also that intensive data curation efforts themselves have been undertaken to make the data suitable for training over by a large neural model, and that the data, despite its size, is still limited. Let us examine some specific, popular datasets and their applications: *BookCorpus* and its companion *MovieBook, Something-Something, HICO* and *HICO-DET*.

### BookCorpus

Zhu et al. (2015) created BookCorpus (a.k.a. the Toronto Book Corpus) as part of a text-to-visual alignment task, aligning sequences in books to sequences in their movie adaptations or equivalent. In the collection process, they eliminated books with less that 20,000 words, however few would argue that a novelette or short story contains less meaningful information than a full-length novel in any regard except quantity. The companion **MovieBook** corpus is a heavily-annotated corpus wherein annotators watched an entire movie with the book open beside it, annotating timestamps down to the second, line numbers, and alignment types. BookCorpus has since gone on to be one of the key datasets that has helped to train large language models like BERT.

### Something-Something

The Something-Something dataset (Goyal et al., 2017), is a well-known dataset in video action recognition, notable for the diversity of actions on display, with a wide variety of objects and situations. The dataset consists of crowd-sourced video snippets all of which are able to be described in the form of *VERBing something* or *VERBing something RELATION something*, where each “something” is replaced with an arbitrary object—anything the video creator happened to have on hand at the time. The videos were crowdsourced with each worker given a prompt of the above form. Workers then filmed the video and logged the objects used in place of “something.” However, beyond minimal quality control like checking for length and removing objectionable content, no second layer of verification of the videos was performed. That is, despite the immense effort expended in the crowdsourcing, there is no evidence that videos were checked to see if they adequately satisfied the prompt, or if a different one of the 174 possible action labels was in fact a better label for a particular video (Patil, 2022).

### HICO and HICO-DET

Chao et al. (2015) introduced HICO, a benchmark for human-object interaction (HOI) detection. While HOI is not a direct mapping to affordances (i.e., not every human-object interaction exploits the object's affordances), it is often a close enough match to be useful. The images in the dataset for each object were first selected from Flickr, and then underwent a rigorous annotation process to verify the presence of a human and the object of interest, and then the presence of any relevant actions related to that object (e.g., “person repairing bike”). HICO's successor dataset, **HICO-DET** (Chao et al., 2018) went even further by extending HICO with object instance annotations that involved not only drawing bounding boxes around the relevant people and objects in an annotated image from HICO, but also annotating links between them so that images containing multiple HOIs have the right humans associated with the right objects.

Of course this type of information encoding is necessary—without it, a neural network could not make sense of the wide distribution of pixel arrangements that could correspond to a *repairing bike* action, or the wide variety of ways that similar actions or objects may be described or depicted.

This is to say nothing of Wikipedia, often regarded as the ultimate free dataset, when in reality it is a massive undertaking by knowledgeable people worldwide, whose construction is explicitly full of structure and metadata meant to make information maximally easy to retrieve. The utility of such datasets cannot be denied, but neither can it be claimed that models trained over such datasets are somehow representation-free.

At the very least, these datasets upon which much of modern AI relies are all weakly annotated. This is not “weakly” in the sense of poorly done, but in the sense of an annotation that is designed to be conducted with the minimum effort possible in order to scale up rapidly, often containing implicit information (such as the sentence pairs used to train BERT's Next Sentence Prediction task, where the “annotation" is simply the pairing extracted from the dataset), which is also often noisy. The job of the large neural network is in part to filter out irrelevant information and discover what exactly the important dependencies are, but nevertheless significant effort has always been expended in making the datasets as friendly to the knowledge extraction process as possible. What this has led to is a cycle of evaluation and benchmarking which is necessary for good comparisons, but also leads to difficulty in applying the conclusions of those comparisons in situations that don't already resemble the training data. Put simply, good performance on ImageNet (or SQuAD, or SWAG, or GLUE, or simulated virtual multimodal shopping cart decision making) does not guarantee equivalent results in real-time human-computer or human-robot interaction, because humans are a constantly moving target, grounding entities in the discourse to items in the world fluently in multiple modalities.

It is well observed that most semantic interpretation is done compositionally. Meaning composition has been called the "holy grail" of cognitive science (Jackendoff, 2002), but if meaning compositionality is to be achieved in machines, what are the means by which meaningful concepts are actually represented? This representation need not be fully explicit, as the aforementioned examples demonstrate, but this also comes at a price where large-scale annotation efforts are required, on the part of researchers or tacitly on the part of the general public, to make enough sense of otherwise unstructured data to make it suitable for machine learning. What the next phase of AI will require is not to eschew representation entirely, but data that is representationally rich and flexible enough to be sample efficient (Stone et al., 2016).

To quote (Dennett, 1993), “*[O]nce we try to extend Brooks' interesting and important message beyond the simplest of critters (artificial or biological), we can be quite sure that something awfully* like *representation is going to have to creep in like the tide*." Such questions became unavoidable during the course of this research. The solution to create a minimal encoding of properties more granular than those typically found in existing datasets has so far delivered promising results in real-time interactive systems, and with methods like transfer learning as demonstrated, we have a way of inferring partial information about new classes from even a small sample of existing classes. Methods for exploiting existing datasets for expanding conceptual vocabulary or situational distinctions are also promising avenues or research, such as augmenting existing HOI datasets to be sensitive to factors like relative orientation (i.e., habitat) and grounding for intent recognition.

## 6. Conclusions and future work

In this paper, we hope to have demonstrated that the notion of situatedness goes well beyond visually grounding a text or a concept to an image or video; rather, it involves embedding the linguistic expression and its grounding within a multimodal semantics that relies on neural and symbolic methods working in tandem to arrive at a more complete interpretation than either alone would provide.

We continue to explore creating stronger links between the habitats and affordances, in order to enable a computer to automatically discover novel uses for an object, such as being able to “poke” with a pencil as you would with a knife, when they are already grasped similarly. Deploying situated grounding-based transfer learning methods such as affordance embeddings live on an interactive agent also raises the prospect of learning affordance and habitat semantics for novel objects through interactions, such as with reinforcement learning.

Neurosymbolic reinforcement learning in a situated grounding context is an ongoing point of study in our work, such as learning to infer novel category distinctions between objects by observing the differences in how they behave under the same conditions and then grounding the learned distinctions to differences in behavior, e.g., what properties of an object enable *stackability* or *rollability*, with preliminary results available in Krishnaswamy and Ghaffari (2022). This work leverages hand-encoded VoxML structures, which are difficult to scale, to nonetheless infer when changes to the environment have occurred, such as when a new type of object has been introduced. By giving the AI agent the capacity to figure out when its own internal model is inadequate and needs to be updated, this allows us to move away from the purely axiomatic reasoning that underlies the frame problem. We have also done preliminary work on expanding neurosymbolic situated grounding methods to the context of real-world robotics (Krajovic et al., 2020), enabling contextual interpretation, dialogue, and question answering in a mixed-reality environment shared by a human and a navigating robot.

This neurosymbolic approach, tightly coupled to a physics-based representation of the world, provides for environmentally-aware models that can be validated; each additional modality supplies an orthogonal angle through which to validate models of other modalities. It provides many methods of encoding context both quantitatively and qualitatively, and provides a model to accommodate both neural and symbolic representations and use them for their different strengths. The diverse types of data available through a situated grounding platform are adaptable to different tasks with novel types of network architectures, with less data overhead than end-to-end neural machine learning. As such, we hope to pose a challenge to the tendency in AI toward increasingly large datasets and bigger models involving more and more parameters, with concomitant costs in energy and resource usage, by utilizing such platforms to provide a sustainable way toward more powerful AI.

## Data availability statement

The datasets presented in this study can be found in online repositories. The names of the repository/repositories and accession number(s) can be found in the article/supplementary material.

## Author contributions

NK was a Ph.D. student and postdoctoral researcher under JP at the time this research was conducted, and continued the research directions as a tenure-track assistant professor in his own lab. JP and NK share joint credit for the development of the VoxML modeling language, on which this work is based. Design of the work discussed in Section 4 was developed by both authors idea of habitats was first introduced by JP and jointly developed by JP and NK. Data was collected and analyzed by NK. Statistical analysis was performed by NK with input from JP. A first draft of the paper was prepared by NK. Both authors wrote all sections of the paper and read, revised, and approved the submitted version.

## Funding

This work was supported by the US Defense Advanced Research Projects Agency (DARPA) and the Army Research Office (ARO) under contracts #W911NF-15-C-0238 at Brandeis University and #W911NF-15-1-0459 at Colorado State University.

## Conflict of interest

The authors declare that the research was conducted in the absence of any commercial or financial relationships that could be construed as a potential conflict of interest.

## Publisher's note

All claims expressed in this article are solely those of the authors and do not necessarily represent those of their affiliated organizations, or those of the publisher, the editors and the reviewers. Any product that may be evaluated in this article, or claim that may be made by its manufacturer, is not guaranteed or endorsed by the publisher.

## Author disclaimer

The points of view expressed herein are solely those of the authors and do not represent the views of the Department of Defense or the United States Government. Any errors or omissions are, of course, the responsibility of the authors.

## Footnotes

^1^Most common systems avoid a nonsensical response with an openly preprogrammed one, e.g., “Interesting question” (Siri) or “You're a great person to chat with!” (Google Mini).

^2^In fact, in extended dialogue exchanges, the model performance drops significantly (Crook et al., 2021).

^3^Other discussion of these developments took place in keynote addresses given at AAAI 2020 by David Cox of IBM, Henry Kautz of the University of Rochester, and Turing Award winners Geoffrey Hinton, Yann LeCun, and Yoshua Bengio.

^4^For example, that a ball is round is taken as a given and so the two words actually collocate comparatively rarely. The result of this can be seen in action by taking two simple sentences: “*This is a* ***ball***” and “*This ball is* ***round****,”* and getting contextualized token embeddings from a text-only model, such as BERT. Using the uncased version of BERT-base, the cosine distance between the two bolded words is 0.377. But if “round” is changed to “flat” or “square,” the cosine distance between the two contextualized embeddings actually goes *down*, to 0.345 or 0.303 respectively.

^5^Data is available at https://github.com/nkrishnaswamy/ underspecification-tests.

^6^A full-length video demo of Diana can be viewed here. More information can be found at https://www.embodiedhci.net.

^7^ https://affordance-embeddings.s3.amazonaws.com/affordance-embeddings.zip

^8^Plain *H* with no reentrancy index refers to a null precondition, meaning that the following behavior is afforded by any habitat or configuration of the object.
